# Variations of cingulate sulcal organization and link with cognitive performance

**DOI:** 10.1038/s41598-018-32088-9

**Published:** 2018-09-18

**Authors:** Céline Amiez, Charles R E Wilson, Emmanuel Procyk

**Affiliations:** Univ Lyon, Université Lyon 1, Inserm, Stem Cell and Brain Research Institute U1208, 69500 Bron, France

## Abstract

The sulcal morphology of the human medial frontal cortex has received marked interest because of (1) its remarkable link with the functional organization of this region, and (2) observations that deviations from ‘normal’ sulcal morphological variability correlate with the prevalence of some psychiatric disorders, cognitive abilities, or personality traits. Unfortunately, background studies on environmental or genetic factors influencing the ontogenesis of the sulcal organization in this region are critically lacking. We analysed the sulcal morphological organization in this region in twins and non-twin siblings, as well as in control subjects for a total of 599 subjects from the Human Connectome Project. The data first confirm significant biases in the presence of paracingulate sulci in left vs right hemispheres in the whole population (twin: p < 2.4.10^−9^; non-twin: p < 2.10^−6^) demonstrating a clear general laterality in human subjects. Second, measures of similarity between siblings and estimations of heritability suggest significant environmental factors, in particular in-womb environment, and weak additive genetic factors influencing the presence of a paracingulate sulcus. Finally, we found that relationships between sulcal organization and performance in cognitive, motor, and affective tests depend on the twin status (Twins versus Non-twins). These results provide important new insights to the issue of the significance of sulcal organization in the human medial frontal cortex.

## Introduction

The sulcal morphology of the human medial frontal cortex is the focus of a good deal of attention for at least three reasons. First, the functional organization of this region is remarkably linked to its sulcal organization^1–4^. Second, a number of studies have suggested that some psychiatric diseases considered as having a neurodevelopmental origin are statistically linked to specific sulcal morphologies, including schizophrenia and obsessive-compulsive disorder^5–13^. Finally, some cognitive abilities^14–17^, as well as different personality traits^18,19^, may be associated with different variations of morphological patterns. These studies suggest that ‘abnormal’ morphological variabilities in this region might be used as endophenotypes of specific diseases, cognitive abilities, and personality traits.

Medial frontal cortex displays strong inter-individual and inter-hemispheric morphological variability. The cingulate sulcus (CGS) is present in 100% of hemispheres. A second sulcus runs parallel to the CGS (the paracingulate sulcus, PCGS), and is present in about 70% of subjects at least in one hemisphere e.g.^1,20,21^. This variability is due to the timing of sulcal ontogenesis: the earlier a sulcus is formed during the development, the weaker the inter-individual and inter-hemispheric variability. Primary sulci appear before the 30^th^ week of gestation (such as the CGS, the central sulcus, etc.) and their presence is constant across individuals. Secondary sulci such as the PCGS appear between the 30^th^ and the 36^th^ week of gestation and show greater variability^22–24^. The most frequent sulcal pattern observed in the cingulate cortex is the presence of a PCGS in the left hemisphere and the absence of a PCGS in the right hemisphere. A number of studies have characterized a pattern of “leftward asymmetry” of the PCGS, where the PCGS is more prominent in the left hemisphere, and smaller or absent in the right hemisphere^1,7,13,20,21,25–27^. This feature of leftward asymmetry correlates with the involvement of the left cingulate cortex in language tasks in right-handed subjects^28^. A reduction in leftward asymmetry has been reported in schizophrenic patients^7–9,29^. PCGS is also less well developed in the left hemisphere of obsessive compulsive disorder patients^10^. In addition, the absence of PCGS has been linked to altered cognitive abilities^13–16,30^ and to specific personality traits^17–19^.

These results might seem surprising given that the absence of a PCGS is not linked to a missing cortical area, but rather to a re-organization of how the cytoarchitectonic areas cover the cingulate cortex^1^. Specifically, in the mid-cingulate cortex, when the PCGS is absent, the ventral and dorsal banks of the CGS are occupied respectively by areas 24c′ and 32′. In contrast, when the PCGS is present, both banks of the CGS are occupied by 24c′ and the PCGS is occupied by 32′^1^. One can, however, hypothesize that although there is no missing cytoarchitectonic area when a PCGS is absent, the axonal tension exerted or the tangential extension of areas may vary depending on whether a PCGS is present. In other words, the sulcal organization might alter the connectivity pattern of this entire region and consequently its function^31^. A better understanding of which factors drive the gyrification processes and lead to inter-individual sulcal variability is therefore required.

Whether the sulcal ontogenesis in the medial frontal cortex is controlled by genetic and/or environmental factors remains unclear^32,33^. The present study aims to first confirm the statistics of cingulate–paracingulate morphologies in a large population, and to estimate whether genetic and/or environmental factors govern the morphological organization of the CGS and PCGS. To this end, we used the standard classification method^27^ to describe the morphology of cingulate and paracingulate sulci in 82 monozygotic twin pairs, 52 dizygotic twin pairs, 67 pairs of singleton (non-twin) siblings, and a group of 197 individual control subjects (a total of 599 subjects) for whom multidimensional data were acquired and provided in the Human Connectome Project database (MGH-USC Consortium, www.humanconnectome.org). Monozygotic twins share 100% of their genotype and dizygotic twins and non-twin siblings both share only 50% of their genotype. In this context, we also assessed the variance of PCGS presence accounted for by genetic and environmental factors using ACE modelling.

## Results

### Statistics on sulcal patterns

#### Patterns of sulci organization

**Figure 1:**
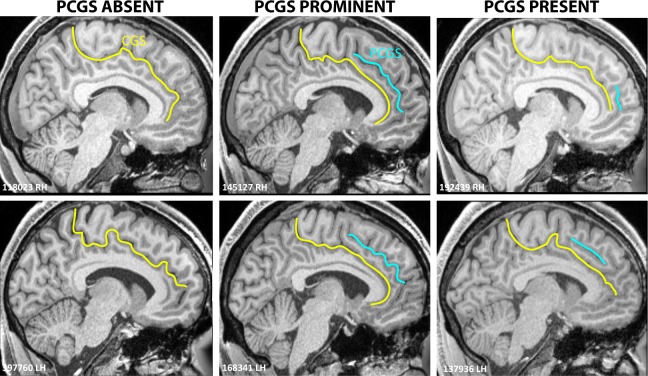
Examples of sulcal morphology in the medial wall. On the left panel, the left (LH) and right (RH) hemispheres of 2 typical subjects displaying only a cingulate sulcus (CGS, yellow). On the middle panel, the left (LH) and right (RH) hemispheres of 2 typical subjects displaying a PROMINENT paracingulate sulcus (PCGS, blue). On the right panel, the left (LH) and right (RH) hemispheres of 2 typical subjects displaying a PRESENT PCGS. Note that a PRESENT PCGS can be observed in the ACC only (top right panel) or in the MCC only (bottom right panel) or at the interface between the ACC and MCC (not shown).

The medial frontal cortex is characterized by the consistent presence of the cingulate sulcus (CGS). In addition, there may be a sulcus running dorsal and parallel to the CGS, called the paracingulate sulcus (PCGS). Both the CGS and PCGS can be continuous or segmented. The PCGS was considered as ‘present’ if the length of one or more segment(s) was ≥2 cm and <4 cm. It was considered as ‘prominent’ if the length of one or more segment(s) was ≥4 cm. It was considered as ‘absent’ if no segment >2 cm was observed. This classification procedure has been developed by Yucel *et al*.^27^ and is the standard in the field (see refs^12,13,27^ for the detailed description of this classification). Fig. 1 displays examples of left and right hemispheres with and without a PCGS.

**Figure 2:**
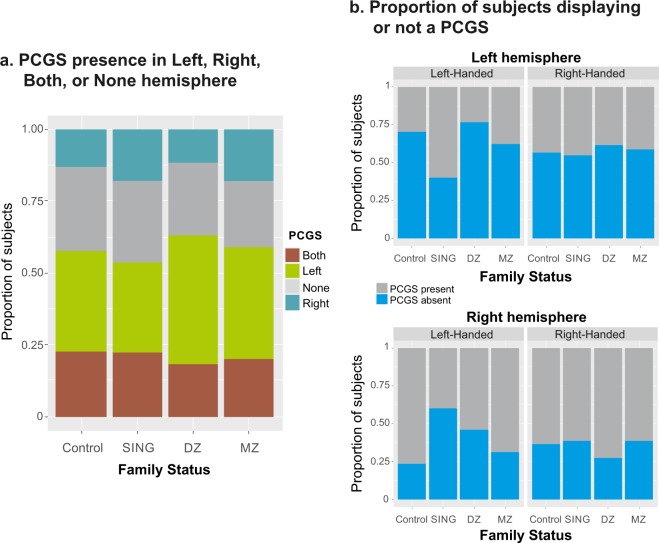
Proportions of subjects with or without a paracingulate sulcus (PCGS). (**a**) Proportion of subjects with no PCGS in either side (none), one pcgs on the right (R) or on the left (L), or a PCGS in both hemispheres (both), and for each population (Control: group of single individuals, SING: group of not twin siblings, MZ: monozygotic twins, DZ: dizygotic twins). (**b**) Proportion of subjects with a PCGS on the left (top) or right (bottom) hemisphere according to handedness and family status. Note the higher incidence of a PCGS on the left hemisphere for both left-handed and right-handed subjects.

Four patterns of sulcal organization were observed in the cingulate cortex based on the simple presence or prominence of a PCGS: 1) in the left hemisphere only, 2) in the right hemisphere only, 3) in both hemispheres, or 4) absent in both hemispheres. These four patterns were observed in the four groups of subjects, as displayed in Fig. 2a. The most frequent pattern was PCGS present/prominent on the left hemisphere and absent on the right (see Fig. 2a). Across all groups a PCGS was observed in the left hemisphere in 58.3% of cases, against 36.2% for the right hemisphere (X-squared = 57.47, df = 1, p-value < 1.10^−10^, 2-sample test for equality of proportions with continuity correction). Taken in isolation, the probabilities to observe a PCGS on the left or on the right hemisphere were both significantly different from the 50% chance level (Left: X-squared = 5.89, df = 1, p-value p = 0.015; Right: X-squared = 4.33, df = 1, p-value p = 0.037, 1-sample proportions test with continuity correction). In summary, the probability of occurrence and imbalance between right and left hemisphere regarding the frequency of PCGS was different from chance level.

**Figure 3:**
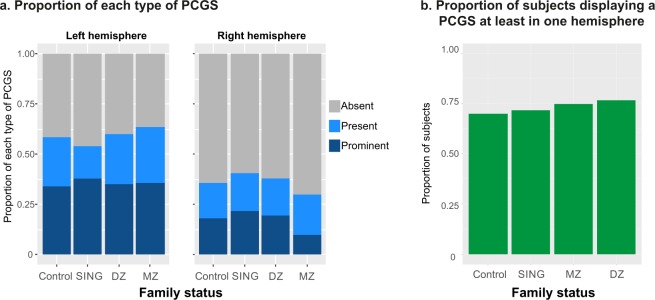
Occurrence of paracingulate sulcus (PCGS). (**a**) Three levels of PCGS are identified: Prominent, Present, and Absent (see Methods). The figure shows the proportion of each case in the different groups of individuals. (**b**) Proportion of individuals with at least one PCGS in one hemisphere.

The PCGS was absent, present, or prominent in the left and/or right hemispheres in the same proportions in the four groups: control (CONTROL group, see Methods), non-twin siblings (SING group), monozygotic (MZ group) and dizygotic (DZ group) twins (see Fig. 3a). One important information is the extension of sulci in the medial wall as it contains dissociable functional areas along the antero-posterior axis. In the four groups, when prominent, the PCGS extended from the ACC to the MCC in the majority of cases (left hemisphere: 97.1%; right hemisphere: 97.1%); when present, the PCGS was located in the majority of cases in the ACC (left hemisphere: 52.1%; right hemisphere: 55.4%). Note however that a present PCGS can also be located in the MCC only (left hemisphere: 25%; right hemisphere: 19.6%) or covering parts of both ACC and MCC (left hemisphere: 22.9%; right hemisphere: 25%). Finally, the proportion of hemispheres displaying a segmented CGS and PCGS did not vary between the four groups (see Table 1; Chi-square, df = 2, *ns* for CGS and PCGS in right or left hemisphere).

**Table 1:** % of hemispheres displaying a segmented CGS and PCGS in the four groups.

Group	Left hemisphere		Right hemisphere	
	CGS segmented	PCGS segmented	CGS segmented	PCGS segmented
Twin MZ	29.3%	35.7%	42.9%	37.5%
Twin DZ	21.8%	26.9%	32.9%	42.2%
SING	33.6%	37.5%	36.6%	27.8%
CONTROL	31%	33.3%	33.5%	32.9%

### Frequency of PCGS

The probability of displaying a PCGS (prominent or present) in the left hemisphere is higher than in the right hemisphere in both twins (MZ + DZ groups, 60.8% in the left vs 39.2% in the right hemisphere) and non-twin (CONTROL + SING groups, 56.2% in the left vs 43.8% in the right hemisphere) populations (twin: X-squared = 35.6, df = 1, p < 2.4.10^−09^; non-twin: X-squared = 22.57, df = 1, p < 2.10^−6^) (Fig. 3a). For a more global evaluation of the frequency of a PCGS in the different populations we estimated the probability of its occurrence in at least one hemisphere, an approach often used in the literature. Figure 3b shows that the percentage of subjects displaying a PCGS at least in one hemisphere is marginally (but not significantly) higher in twins -whether monozygotic (76.8%) or dizygotic (75%)- than in the two groups of non-twin subjects (71.6% and 70.1% in SING and CONTROL respectively) (Twins vs. Not Twins, X^2^ = 1.94, df = 1, p < 0.16). Note that the values observed for control subjects is the closest to those reported in the literature. In conclusion, the overall likelihood of having a PCGS in twins is not significantly different than in other subjects.

According to Yücel *et al*.^27^, one main characteristic of the inter-hemispheric variability of PCGS organization is a leftward asymmetry which is somewhat reflected in the above description. Such leftward asymmetry was defined as brains displaying 1) a prominent PCGS in the left hemisphere and a present or an absent PCGS in the right hemisphere, or 2) a present PCGS in the left hemisphere and no PCGS in the right hemisphere). Using this definition, we observed that leftward asymmetry was more frequent in twins (MZ + DZ) (47.4% of subjects) than in non-twins (38.4% of subjects) (2-sample test for equality of proportions with continuity, X-squared = 4.57, df = 1, p < 0.032). By contrast, the rightward asymmetry is observed similarly in twin (17.5% of subjects) and non-twin (18.1% of subjects) populations (X-squared = 0.006, df = 1, ns).

#### Handedness, gender, and twin status effect on sulci organization

We assessed the handedness, gender, and twin status (i.e. Twins versus Non-Twins, see Methods) effects on the leftward PCGS bias. Groups MZ TWIN, DZ TWIN, SING, and CONTROL contained respectively, 90.2%, 87.5%, 92.5%, and 91.4% of right-handed subjects. The probability of a PCGS in the left hemisphere or in the right hemisphere was similar in both left-handed (X-squared = 0.66, df = 1, p < 0.5, ns) and right-handed subjects (X-squared = 0.003, df = 1, p < 0.96, ns) (Fig. 2b).

We first evaluated whether the probability of a PCGS in at least one hemisphere was modulated by subjects’ gender, handedness, or by twin status. Results show that none of these parameters influenced the probability to observe a PCGS at least in one hemisphere (binomial GLM, twin status x gender x handedness, all p > 0.05).

We then assessed whether the probabilities to observe a segmented CGS/PCGS in both hemispheres were influenced by subjects’ gender, handedness, or by twin status. Results show that the probability of observing a segmented CGS in the right hemisphere was only slightly influenced by an interaction between handedness and gender (estimate = 1.3274, std error = 0.5960, Z = 2.227, p < 0.025). There was no influence of handedness and/or gender on the presence of segmentation in either the CGS in the left hemisphere or the PCGS bilaterally depending on the twin status (Twins versus Non-Twins) (Logistic regression, ns).

### Impact of CGS/PCGS morphology and twin status on cognitive, motor, and emotional tests

Several studies have observed statistical links between specific cognitive abilities^14–17^ or personality traits^18,19^, and variations of morphological patterns. The HCP database provide a unique opportunity to study these relationships in the case of siblings. We first analysed whether selected behavioural test scores where different between Twin and Non-Twin groups. Scores were first cleaned from outliers as described in the Method section. Because some scores displayed non-parametric distributions (i.e. Short Penn Continuous Performance Test -SCPT_FN-, Mini Mental State Examination -MMSE- Score, Delay Discounting -DDisc_AUC_200), both ANOVA and Kruskal-Wallis statistical tests were applied. In both cases, results showed that the scores were identical across the 2 groups (ANOVA and Kruskal-Wallis rank sum tests at p < 0.05).

We then assessed whether the probability to observe a PCGS at least in one hemisphere had some relationships with behavioural scores in cognitive, motor, and affective tests depending on the twin status by fitting a binomial GLM with PCGS as dependent variable and incorporating the twin status factor interacting over all test scores. This basically tested whether any of the test scores would relate to the presence of a PCGS at least in one hemisphere differently for the Twin and Non-Twin populations. We tested the full model and also applied a model selection. Data revealed that 5 scores displayed some differential values based on the probability to have a PCGS at least in one hemisphere and depending on the twin status: Mini Mental State Examination -MMSE-, Card sorting -CardSort-, Endurance, Fear Affect -FearAffect-, and Fear somatic -FearSomat- scores. A main effect was found for MMSE score and the interaction with twin status was only marginally significant (GLM interaction term zygosity status × MMSE score: z = 1.933 p < 0.053). All other scores revealed significant interactions. Specifically, results showed differential relationships between the probability to observe a PCGS at least in one hemisphere and test scores for the Twin and Non-Twin groups (Twin status × CardSort: estimate = −0.086, standard deviation = 0.029, z = −2.999, p < 0.003; Twin status × Endurance: estimate = −0.035, standard deviation = 0.016, z = −2.198, p < 0.05; Twin status × FearAffect: estimate = −0.11, standard deviation = 0.032, z = −3.29, p < 0.001; Twin status × FearSomat: estimate = 0.078, standard deviation = 0.031, z = 2.510, p < 0.02). Statistical models showed that the relationship with Fear Affect and MMSE scores were significant only for the Non-Twins population (z = 2.93, p < 0.005 and z = −2.37, p < 0.05, respectively for FearAffect and MMSE), and that endurance scores effect was significant only for the Twins population (z = −1.96, p < 0.05).

**Figure 4:**
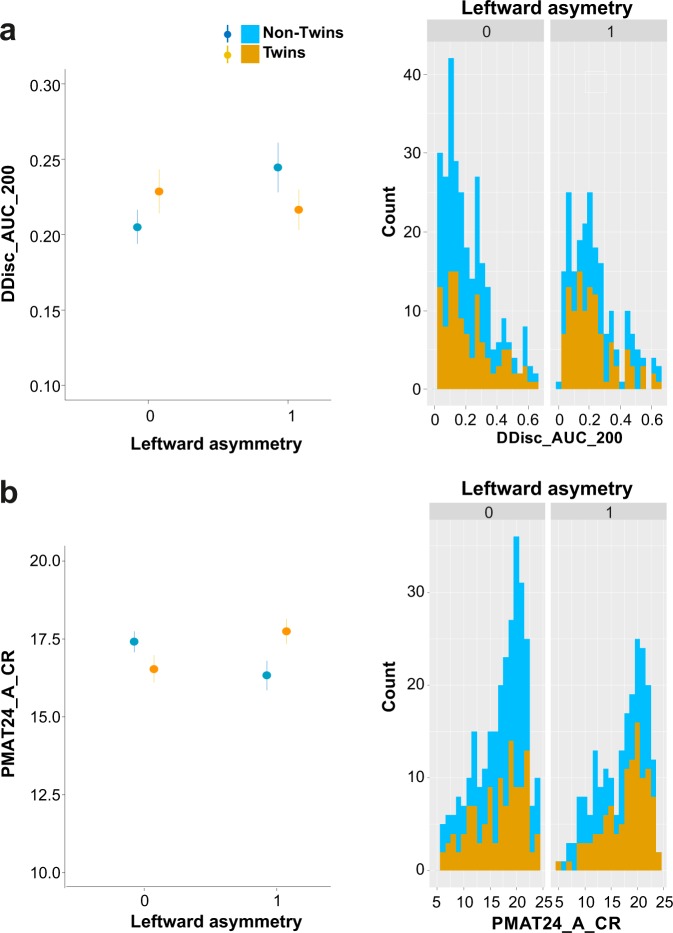
Average scores in the PMAT24 (**a**) and DDisc (**b**) tests in the Non-Twin and the Twin populations displaying a leftward asymmetry (left panels). On the right panels are displayed the distributions (count) of scores for the Non-Twin and the Twin populations displaying a leftward asymmetry.

Finally, we assessed whether the leftward or rightward asymmetry of the organization of the CGS/PCGS modulated tests scores. In that goal, analyses were performed separately for rightward and leftward asymmetry (note that, as for the analysis above, PCGS was also entered as dependent variable). None of the tests scores correlated with the rightward asymmetry (logistic multiple regression, at p < 0.05). By contrast, the leftward asymmetry impacted scores in Penn Progressive Matrices -PMAT24_A_CR- test and DDisc_AUC_200 scores, but the modulation was inversed in Twins and Non-Twins (see Fig. 4). Note that these scores refer respectively to number of correct trials in non-verbal reasoning matrices (Raven’s matrices) and to the area under the curve measured in a delay discounting test (see Methods). Specifically, concerning PMAT24_A_CR scores (Fig. 4a), the average scores for Twins displaying a leftward asymmetry was 17.75 against 16.54 for Twins displaying no leftward asymmetry (i.e. a difference of +1.21); average score for Non-Twins displaying a leftward asymmetry was 16.33 compared to 17.41 for Non-Twins displaying no leftward asymmetry (i.e. a difference of −1.08). Note that the scores across the whole population range from 5 to 24 correct trials. Regarding DDisc_AUC_200 scores (Fig. 4b), the average discounting score for Twins displaying a leftward asymmetry was 0.216 against 0.228 for Twins displaying no leftward asymmetry (i.e. a difference of −0.012). By contrast, the average score for Non-Twins displaying a leftward asymmetry was 0.244 against 0.205 for Non-Twins displaying no asymmetry (i.e. a difference of +0.039). Note that the DDisc scores range from 0.015 to 0.664 in the whole population. DDisc scores correspond to the area under the discounting curve. The steeper the discounting (i.e., the lower the subjective value of delayed rewards), the smaller the score value.

### Similarity of cingulate morphology in monozygotic twins compared to dizygotic twins and non-twin siblings

The mere presence of a PCGS (present or prominent) does not seem to vary with the family status. However, one can assess whether the similarity of the pattern presence/absence of PCGS in the left and right hemisphere between siblings, is influenced by the genetic status. Indeed, dizygotic twins and non-twin siblings both share 50% of their DNA, as opposed to monozygotic twins who share 100% of their DNA.

**Figure 5:**
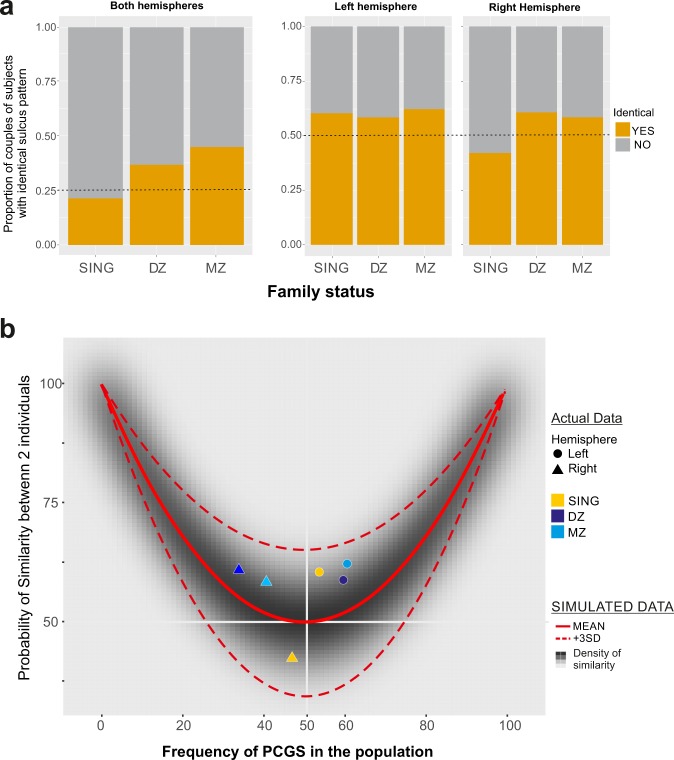
Similarity of patterns in the medial wall in siblings. (**a**) Proportion of cases with identical patterns (with or without a PCGS) for both hemispheres (left panel) and individually for the left and right hemispheres (panels on the right). Data are presented for the 3 groups of siblings. Note a higher occurrence of similarity for monozygotic twins, which is particularly related to a higher similarity for the right hemisphere. (**b**) Simulations. Frequency of similarity between the hemispheric pattern of 2 randomly sampled individuals in simulated populations with various basal frequencies of sulcus occurrence. At 0 percent of sulcus in the population the probability to find similar patterns (no sulcus) in the population is on average maximal (100%). Same for a population in which a PCGS would be present in 100% of cases. If the likelihood of a PCGS is at 50% (x axis) then the probability of having two individuals with the same pattern in the same hemisphere is 50%. At that level, the variance is maximal. The mean probability of similarity is indicated with the red curve. Real data from the human database is represented by single points overlapped on the simulation to show their relationship to randomly generated data. Disks and triangles represent data (percent of similarity) for the left and right hemispheres respectively. Colors represent the different groups of siblings.

We analysed data from couples of twins and non-twin siblings (i.e. MZ, DZ, and SING groups). We first assessed the level of similarity between hemispheres (e.g. if a PCGS is present in the left hemisphere and absent in the right hemisphere in one sibling, is it also the case in the other sibling?). The chance level for the 2 hemispheres to be identical in 2 subjects is 0.25. Hemispheres taken together were apparently more alike in monozygotic (45.1%) than in dizygotic siblings (37.3%) or non-twin siblings (21.2%) (Fig. 5a-left). However, the proportion of both hemispheres similarity in MZ and DZ twins did not differ (X^2^ = 0.51, df = 1, p-value = 0.47) but differed from non-twin siblings (MZ: X^2^ = 8.23, df = 1, p-value < 0.005. DZ: X^2^ = 2.90, df = 1, p-value = 0.088). Taken as a whole these proportions are significantly different from what is expected by chance (X^2^ = 9.29, df = 2, p < 0.01), and they were individually different from 25% chance level for MZ twins (X^2^ = 16.65, df = 1, p-value < 1e-04), marginally different in DZ twins (X^2^ = 3.45, df = 1, p-value = 0.062), and not different from chance in non-twin siblings (X^2^ = 0.32, df = 1, p-value = 0.56).

For separate hemispheres (Fig. 5a-right), only the similarity of the left hemisphere in MZ subjects was more frequent than 50% chance (X^2^ = 4.40, df = 1, p-value = 0.035).

In assessing the probability that 2 individuals from a given population (for example DZ twins) have identical left or right hemispheres, we must take into account the baseline frequency of sulcus occurrence for that population. To illustrate this point, we computed the likelihood of finding similarity between any 2 individuals by chance (i.e. without any genetic or active environmental factor of influence), across all virtually possible sulcus frequencies. The simulated cases are presented in Fig. 5b. The predicted average similarity is displayed by the solid red line in Fig. 5b (calculated on 1000 permutations for each case of sulcus proportion). This line tells us the average probability of similarity we should expect by random for each frequency of sulcus, and we can compare this line to the actual data. The inverted U shape of the curve simply reflects the fact that very low or very high frequencies of a sulcus in a population increases the probability of finding similarity between two unrelated hemispheres. The data from the database are the coloured points on this figure. First note the clear Left (disks) / Right (triangles) asymmetry detailed above for the probability of presence of a PCGS. The measured proportions of similarity between left hemispheres are shown as coloured discs, and they are all above the mean simulated data in Fig. 5b. These points are, however, mostly within the range of values of similarity obtained across permutations (transparency reflects density of points; dashed red lines represent the +/−3 SD limits around the mean).

We can perform the same comparison for the right hemisphere. All probabilities of similarity were again within the range of mean +/−3sd of the simulated data, although the SINGLE data revealed a different trend compared to Twins (yellow triangle). Overall the statistics suggest that some properties might be influenced by environmental (e.g. in womb) factors and a small fraction might be transmitted genetically (left PCGS in MZ).

### Heritability of PCGS: ACE models

The above descriptive and global analyses suggest little influence of genetic factors on the presence of PCGS in the medial wall. To specifically address the question of heritability of the presence of a PCGS we performed ACE path analyses. The theoretical framework behind these analyses states that the variance of a character in a population is linked to the sum of variances from additive genetic effects (A), from shared environment effects (C), and from unique or unshared environment effects (E)^34^. One key element for such approach is to compare a phenotypic concordance in MZ and DZ twins that have different proportions of identical alleles. Although the approach has some drawbacks it allows the use of complete statistical modelling. The analyses were performed using structural equation modelling and path model specifications to estimate the contributions of latent variables (A, C, and E) to the variance of the observed variable (here presence of a PSCG). The statistical models (see methods) were fit on binary data to study separately the presence-absence of PCGS in the left or right hemispheres in the MZ and DZ pairs of subjects. For both hemispheres, we found that the major part of variance was explained by the unique environment component (E): 66% and 81% for left and right hemispheres respectively. The shared environment accounted for 0.02% and less than 0.001% for left and right hemispheres respectively. Finally, the additive genetic component, that can be associated to a heritability factor, accounted for 34% and 19% of the variance in the PCGS presence in the left and right hemispheres. The imbalance between right and left hemispheres confirms the descriptive observation of similarity reported above.

To test whether the different parameters of the ACE models were contributing significantly, we compared with simpler nested models removing the contribution of A and C. In all cases, the simpler model including only the latent variable E for unique environmental variance was equally efficient than the ACE, AE or CE models (Log likelihood ratio tests; all p > 0.05). The ACE vs E model comparison for the left hemisphere revealed a marginal non-significant difference (Diff in LL = 4.45, p = 0.11).

## Discussion

The analyses of the presence of the PCGS in twin and non-twin subjects from the Human Connectome project database have 5 main outcomes. First, the likelihood of a PCGS is higher in the left hemisphere than in the right hemisphere and with statistics higher than chance levels, suggesting factors influencing the laterality in all humans. However, the leftward asymmetry of PCGS was more frequent in twins (MZ + DZ) than in non-twins. Second, the proportion of similar hemispheres did not differ between MZ and DZ twins but differed from non-twin siblings. Both results suggest the influence of weak genetic and stronger environmental factors. Third, statistical estimation of heritability with MZ and DZ twin data did not reveal significant additive genetic effects on the occurrence of a PCGS in left or right hemispheres. Fourth, results revealed complex relationships between the morphology of CGS/PCGS and performance in cognitive, motor, and affective tests depending on the twin status (Twins versus Non-twins). Finally, our study shows that several factors govern the ontogenesis of the sulcal organization in the medial frontal cortex, and differentially for the two hemispheres.

The finding that two twins have a higher probability to display similar sulcal organizations for both hemispheres supports a genetic influence on gyrification in the medial wall^32,33,35–38^. However, the proportion of similar hemispheres is not different between MZ and DZ twins. The leftward asymmetry was higher in twins (MZ and DZ) than in non-twins, suggesting a prevalence of left PCGS in couples which had a common womb environment. Regarding sulcus emergence and gyrification, the current understanding is that primary sulci are more predetermined than secondary sulci, the presence of the latter being influenced by environmental factors^39,40^. Lohman *et al*.^39,41^ have shown that the overall folding pattern of the deepest and earliest formed sulci (primary sulci) is well preserved in MZ twins, and is therefore under genetic control (for review, see Fernandez *et al*.^42^). Our findings suggest some predetermined driving influence for a secondary sulcus, the PCGS. First, the increased probability of PCGS in the left hemisphere and decrease of probability in the right hemisphere compared to chance in all human subject groups suggest a yet-to-be-described human trait that is likely to have some genetic basis. However, additive genetic effects estimated from the ACE model using MZ and DZ twin data gave no indication of an influence of additive genetic factors. Note, incidentally, that the models led to a higher estimated additive genetic variance for the left hemisphere than for the right (34% and 19%). These last results must be taken with caution as ACE models, although simple and practical models to estimate heritability, rely on several strong assumptions and miss several potentially important factors of influence^43^.

As mentioned above, some evidence suggests in-womb environmental influences as revealed by differences between twins and non-twin subjects. Figure 4b shows that the pattern of twin populations (blue/purple points) diverge from the chance level with a higher level of similarity for a PCGS on the right compared to non-twin subjects (yellow points). This, together with the significant differences in leftward asymmetry, provide some evidence for the proposition that environmental factors also influence the gyrification process. Specifically, twins may display different cortical folding than non-twins because of the presence of two foetuses in the uterus, driving the emergence of a secondary sulcus in the form of the PCGS^44,45^. However, this effect would be differentially expressed in the two hemispheres, and other environmental factors might need to be taken into account.

The relationship between CGS/PCGS morphological patterns and the measures of motor and cognitive performance included in our analyses revealed complex relationships depending on the twin status and in some test reverses relationships. First, whereas the presence of a PCGS at least in one hemisphere impacted the scores in MMSE cognitive test and Fear affect test only in Non-Twins population, this morphological pattern impacted the scores of endurance only in the Twins population. Second, whereas Twins displaying a leftward asymmetry have better scores in the Penn Progressive Matrices cognitive test (PMAT24_A_CR scores) than Twins displaying no asymmetry, Non-Twins displaying this asymmetry have lower scores in this test than Non-Twins displaying no asymmetry. The opposite was found in the cognitive Delay Discounting test (DDisc_AUC_200 scores): Twins displaying a leftward asymmetry have lower scores than Twins displaying no asymmetry, Non-Twins displaying this asymmetry have higher scores than Non-Twins displaying no asymmetry. Note that these inverse effects corresponded to relatively small differences in average scores. Our results therefore call into question to what extent structural variability of the cingulate cortex may directly influence its function and dysfunction. Indeed, our results strongly suggest that the use of the CGS/PCGS morphology as a biomarker for certain characteristics requires a-priori knowledge about the twin status of subjects. The presence of a PCGS has been correlated with better high-order cognitive abilities^14,16,30,46^. But our analyses provide new evidence that sulcal organization in medial frontal cortex relates differently to the various functions studied in the database, across motor and cognitive domains, depending on the twin status. These include performance in tasks associated with robust fMRI activation in the region (e.g. PMAT24). Further, results confirm the leftward asymmetry of the PCGS in all groups and show that this asymmetry is not modulated by handedness and gender. Therefore, the leftward asymmetry might not be due to the lateralization in the left hemisphere of language areas as previously suggested^28^. These negative results should be treated with the caveats associated with such outcomes. Notably, we are using battery tasks, rather than the specific tests used in the studies cited above. Nevertheless, we have analysed a significant number of subjects (indeed more than the studies above combined) with a well-respected database, and our results suggest that correlation of the sulcus morphological patterns with cognitive abilities varies depending on the twin status. It is possible that such measurements are also strongly dependent on the pool and number of subjects assessed. It is also reasonable to think that these measurements may depend on the location of the PCGS. Indeed, we have shown that the PCGS can be present only in the ACC or only in the MCC, or it can extend from the ACC to the MCC when it is prominent (see results). Studies have repeatedly shown that the ACC and the MCC are functionally different (for review, see Vogt 2016^47^) and future studies should therefore take into account the location of the PCGS. Notably if the presence/absence of a PCGS would influence cognitive functions related to specific MCC regions involved in adaptive cognition then it is expected that this would concern cases where PCGS morphological variance concerns the corresponding MCC region. Finally, discrepancies with previous studies might be due to the statistical analysis. In studies assessing relationships between PCGS/CGS morphology and cognitive or motor functions, the morphology is usually considered as an independent variable whereas here it was considered as a dependent variable, which allowed us to address complex relationships with zygosity and cognitive and motor abilities.

The relationship between sulcal variability of the cingulate cortex and neurodevelopmental pathologies, cognitive processes, and personality traits could be explained by the theory of cortical gyrification driven by white matter fibres tension-based mechanisms^31^. More tension may therefore be observed when a PCGS is present, which could modify neuronal or synaptic density and cortico-cortical connectivity^31^. This would be linked to varying neurodevelopmental processes that might be at the origin of psychiatric diseases, different cognitive abilities, and various personality traits. However, this tension-based hypothesis has been recently challenged^44,48^ suggesting that the axonal tension is not directly linked to the gyrification process (for review, see refs^38,42^). Other key factors combined like differential expressions of transcription factors and variance in cell proliferations during brain development might contribute to variance in cortical morphology^49^.

In the present study, the ACE model does not distinguish between prenatal and postnatal environments. The impact of post-natal environment on sulcal organization cannot be assessed because the HCP database does not provide information relative to the post-natal subjects’ living environment. However, since the CGS is a primary sulcus and the PCGS is a secondary sulcus (see introduction), they are formed before birth. Therefore, it is unlikely that post-natal environmental factors may influence the setup of the sulcal morphology within this region. This conclusion is supported by a recent study showing that post-natal environmental factors have a limited effect on the depth of sulcal pits, which are the cortical folds appearing first during gestation^50^. This is also supported by studies suggesting that prenatal environment have long-term effects across the life-span on cognitive abilities in both twins and non-twins^51–53^.

To conclude, the morphology of the medial wall and specifically the existence of a PCGS is driven by multiple factors, as PCGS laterality seems to be a species-specific trait showing variations in the population, and with weak additive genetic effects as suggested by twin data. The statistical laterality of the PCGS is a very stable and reproducible morphological trait at the level of populations. Finally, correlations between the sulcal pattern and motor, cognitive, and affective abilities are complex and are modulated by the twin status.

## Methods

### Subjects

We studied high-resolution anatomical scans of 641 subjects in total. Acquisition parameters of T1 anatomical scans are the following: whole head, 0.7 mm^3^ isotropic resolution, TR = 2.4 s, TE = 2.14 ms, flip angle = 8° (more details can be found on https://humanconnectome.org/storage/app/media/documentation/s1200/HCP_S1200_Release_Appendix_I.pdf). All T1 scans were registered in the MNI stereotaxic space. The presence or absence of a PCGS was assessed on these normalized brains.

Analysis of anatomical T1 scans were performed in four groups:

- MZ GROUP: 82 monozygotic twin pairs (30/52 M/F)
- DZ GROUP: 53 dizygotic twin pairs (19/33 M/F). Note that the subjects in each pair in the DZ group are all of same sex. Indeed, most of the twin population recruited in HCP were recruited from previous twin studies that specified same gender twins (MZ or DZ) because of potential gender confounds in making comparisons between twins and to avoid sex being a confound for heterozygosity.
  - SING GROUP: 67 pairs of singleton (non-twin) siblings (73/61 M/F)
  - CONTROL GROUP: 197 subjects with no sibling in the database (93/104 M/F)

All subjects were participants of the Human Connectome Project (HCP) (humanconnectome.org). The participants in the HCP study were recruited from the Missouri Family and Twin Registry that includes individuals born in Missouri^54^. The age of monozygotic twins, dizygotic twins, singleton siblings, and control subjects was, respectively, 30 ± 3.3 years, 29.2 ± 3.4 years, 28 ± 3.7 years, and 28.8 ± 4 years. Note that the monozygotic twin pairs contain 75.7% of females. This bias in the database is coherent with some reports of higher survival rates for female monozygotic twins.

The full set of inclusion and exclusion criteria is detailed elsewhere^54^. In short, the HCP subjects are healthy individuals who are free from major psychiatric or neurological illnesses. They are drawn from ongoing longitudinal studies^54–56^, where they received extensive previous assessments including the history of drug use, emotional, and behavioral problems. The experiments were performed in accordance with relevant guidelines and regulations and all experimental protocol was approved by the Institutional Review Board (IRB) (IRB # 201204036; Title: ‘Mapping the Human Connectome: Structure, Function, and Heritability’). All subjects provided written informed consent on forms approved by the Institutional Review Board of Washington University in St Louis. In addition, the present study received approval (n°15-213) from the ethic committee of Inserm (IORG0003254, FWA00005831) and from the Institutional Review Board (IRB00003888) of the French institute of medical research and health.

#### Assessed functions

In order to assess the correlation between morphology and functions known to depend on the cingulate cortex, we analysed the scores of the different groups of subjects in the following behavioural tests (see below for more details):

**Motor functions:**

- **Dexterity** (Dexterity_AgeAdj in the database): A test of manual dexterity, measured in the time taken by participants to accurately place and remove 9 plastic pegs on a plastic pegboard. Participants have a practice trial and then the timed trial with each hand. The measure is the time in seconds to complete the task with the dominant hand. It is adjusted for age.
- **Endurance** (Endurance_AgeAdj in the database): A test of physical endurance, it is adapted from the American Thoracic Society’s 6-Minute Walk Test Protocol. The measure is the distance a participant can walk on a 50-foot long course during a 2-minute period. A score is calculated in feet and inches. It is adjusted for age.
**Cognitive functions:**
- **Alertness** (MMSE_Score in the database): An evaluation of cognitive abilities and mnemonic performance. The measure replicates the Mini Mental State Examination or Folstein’s test (1975)^57^.
- **Episodic Memory** (Picture Sequence Memory, PicSeq_AgeAdj in the database): A test of recall, participants are presented with increasingly lengthy series of illustrated objects and activities. The participants must recall the sequence of pictures presented in two learning trials. The measure is the number of adjacent picture pairs participants correctly place in sequence, up to the maximum value for the sequence, which is one less than the sequence length. So if pictures in locations 9 and 10 are placed in that order and adjacent to each other anywhere – such as slots 1 and 2 – one point is awarded. If there are 18 pictures in the sequence, the maximum score is 17, as there are 17 possible adjacent pairs. The scores are adjusted for age.
- **Executive Function/Cognitive Flexibility** (Dimensional Change Card Sort, CardSort_AgeAdj in the database). A measure of cognitive flexibility. Participants must take start from two initial target pictures and match a series of subsequent pictures which might match along one of two dimensions (e.g., shape and color). Participants will initially match along one dimension (e.g., color). After a number of trials, they must change and match according to the other dimension (e.g., shape). In “Switch” trials the participant must change the dimension being matched. So having matched to shape for 4 straight trials, the participant may be asked to match on color on the next trial and then return to shape matching. The task hence requires the cognitive flexibility necessary to move efficiently between rules. The measure is derived form a combination of accuracy and reaction time. It is adjusted for age.
- **Executive Function/Inhibition** (Flanker Task, Flanker_AgeAdj in the database). A test of attention and inhibitory control. Participants must focus on a target stimulus while inhibiting their attention to flanking arrow stimuli. The target stimulus may bet congruent with the flankers by pointing in the same direction, or be incongruent. The measure is derived form a combination of accuracy and reaction time. It is adjusted for age.
- **Fluid Intelligence** (Penn Progressive Matrices, PMAT24_A_CR in the database). A test of fluid intelligence. This directly uses Raven’s Progressive Matrices^58–62^, and specifically Form A of the abbreviated version developed by Gur and colleagues^63^. Participants are presented with patterns made up of 2 × 2, 3 × 3 or 1 × 5 arrangements of squares, with one of the squares missing. The participant has five options from which to pick the response that best completes the pattern in the missing location. The task has 24 items and 3 bonus items, arranged in order of increasing difficulty. The task discontinues if the participant makes 5 incorrect responses in a row.
- **Self-regulation/Impulsivity** (Delay Discounting: DDisc_AUC_200 in the database). A test of impulsivity, the delay discounting paradigm measures the phenomenon of undervaluing of rewards that are delayed in time. This task identifies ‘indifference points’ at which a participant is equally likely to choose a sooner but smaller reward (e.g., $100 now) versus a later but larger reward (e.g., $200 in 3 years). The approach follows Green and Myerson^64,65^: delays are fixed and reward amounts are adjusted on a trial-by-trial basis based on the participants’ ongoing choices. This allows rapid identification of indifference points. This approach provides reliable estimates of delay discounting^64^. We extract a summary measure in the form of an area-under-the-curve discounting measure (AUC). This is a valid and reliable index of how steeply individual discounts delayed rewards^66^.
- **Sustained Attention** (Short Penn Continuous Performance Test, SCPT_FN in the database). A test of attention, this applies the Short Penn Continuous Performance Test (Number/Letter Version)^67,68^. Participants must observe vertical and horizontal red lines as they flash up on a computer screen. Subjects must respond when the lines form either a number or a letter. The number/letter rule is changed between blocks. Lines are displayed for 300 ms followed by a 700 ms ITI.

**Emotional functions - Negative Affect (Sadness, Fear, Anger):** The emotional tests assess two specific aspects: 1) The NIH Toolbox Fear-Affect Survey (FearAffect_Unadj in the database) uses items from the PROMIS Anxiety Item Bank. The measure is self-reported fear and anxious misery. 2) The NIH Toolbox Fear-Somatic Arousal Survey (FearSomat_Unadj in the database) provides a 6-item calibrated scale comprised of items from the Mood and Anxiety Symptom Questionnaire. It assesses somatic symptoms related to arousal. Scores are not corrected for age.

#### Statistical analysis

GLM analyses were applied to test the impact of 1) the zygotic/sibling status of subjects on the organization of the CGS/PCGS, and 2) the behavioural tests that are known to depend on the cingulate cortex (see above). Further GLM analyses were performed to assess the impact of the handedness or gender on the organization of the CGS/PCGS depending on the Twin status: Twins (including MZ and DZ groups) and Non-Twins (including CONTROL and SING groups).

Concerning the analysis of the impact of the organization of the CGS/PCGS impact on behavioural tests, we first tested for the presence of multicollinearity. In that goal, we used the Variance Inflation Factor (VIF) calculated on a GLM with all scores as fixed effects. Although 2 factors (CardSort_AgeAdj and Flanker_Unadj) were significantly correlated (R^2 = 0.24, p < 0.001), none of the VIF were higher than 2, a limit of 4 being usually taken as problematic. Analyses of relationships between morphology and behavioural scores were performed using logistic regressions following the successive steps: model fit, model selection, and deviance analysis. For instance, for the analysis of PCGS in one hemisphere we fitted the following model: PCGS_in_One_Hemisphere = ${\beta }$_0_ + ${\beta }$_1_.Twin_Status * (${\beta }$_2_.Flanker_AgeAdj + ${\beta }$_3_.MMSE_Score + ${\beta }$_4_.CardSort_AgeAdj + ${\beta }$_5_.PMAT24_A_CR + ${\beta }$_6_.DDisc_AUC_200 + ${\beta }$_7_.Dexterity_AgeAdj + ${\beta }$_8_.Endurance_AgeAdj + ${\beta }$_9_.PicSeq_AgeAdj + ${\beta }$_10_.SCPT_FN + ${\beta }$_11_.FearAffect_Unadj + ${\beta }$_12_.FearSomat_Unadj) + ɛ. The investigation of links between anatomical traits and behavioral data requires behavioral variability and temporal stability. We therefore first verified that the distributions of all the behavioral measures assessed in the present paper were not presenting ceiling effect. This analysis revealed that the distributions of several scores had significantly skewed distributions (Flanker, MMSE, DDiscount, SCPT, and Fear Somatic; skewness test at p < 0.05) and were therefore not presenting ceiling effect. Regarding temporal stability, because the HCP database contains at the current time only a very limited number of re-tested subjects, this effect of this aspect on our set of data cannot be interpreted or discussed.

Linear models can be sensitive to outliers, even in large samples. Although the identification of so-called outlier values are debated and complicated matters, we also fitted all the statistical models (behavioral measures and cognitive scores) used in this paper without extreme values of predictors. We reasoned that if, by removing extreme points in predictors, the linear model fits would change significantly then this would be an indication of biases. In that goal, for each predictor in our statistical models, we used Tukey’s method to identify the outliers ranged above and below the 1.5*IQR – interquartile range). Pairs plot were also used to verify the existence of outlier values. We then removed subjects with outlier values for at least one of the predictor. With this procedure, 497 subjects were kept. We then ran all models as in the analysis including the outliers (see above). Finally, we ran stepwise model selections on the different full models. Importantly, because the results were identical than those obtained while including the outliers, we present only results obtained in the analysis excluding the outliers.

Note that 1) the handedness has been transformed into a binomial factor Hand [Left/Right] to simplify the analysis, and 2) in order to incorporate the cognitive score tests in generalized linear models we standardized the score values by de-meaning and dividing by standard deviation.

All statistics were performed with R software, R Development Core Team^69^ under R-Studio^70^.

#### Simulation

The probability to have identical hemispheres, e.g. same left hemisphere with or same without a PCGS, between two independent populations depends on the base proportion of that sulcus in the population. To estimate the probability distribution of similarity expected from random populations, we generated, two random samples corresponding to vectors of 0 and 1 s corresponding to with or without PCGS, with the same virtual probability of having a sulcus, and measured the proportion of similarity. We permuted the 2 samples 1000 times to get the distribution of similarity proportions. This was performed for each frequency of sulcus from 0 to 100%. Mean and standard deviations were obtained for each virtual sulcus frequency.

#### ACE model and structural equation modelling

In order to study whether the variance of presence of a PCGS in a hemisphere could be explained by genetic factors, we used Structural Equation modelling to analyse the twin data within the framework of ACE models (A = additive genetic effects, C = shared environment effects, E = unshared environment effects). The idea is to compare the phenotypic concordance of monozygotic (MZ, identical) twins versus dizygotic (DZ, fraternal) twins. Analyses were performed in R using the OpenMX^71^ and umx library^72^.

The occurrence/absence of a PCGS (binary factor) in the left or right hemisphere were the observed variables. We use a common path diagram for the ACE model^73^, with latent variables (A, C, E) for each observed variable (T1 and T2, one per member of a couple). In the path diagram, the correlation between the latent variables A1 and A2 for MZ twins was set to 1, and set to 0.5 for DZ twins. In both DZ and MZ, the path between C1 and C2 was set to 1. The main paths from latent variables to the respective observed variables were free parameters and estimated by fitting the model. To model the ordinal raw data, we defined thresholds using the umx library. We obtained from each model, parameter estimates (variance for A, C and E variables), standardized variance components (components divided by total variance), and AIC.

To evaluate the significance of each of the model parameters, we fit nested submodels in which the parameters of interest were set to zero. Hence, in addition to the ACE model, CE, AE, and E models were fit and compared using log-likelihood ratio tests.
